# Effect of miR-149-5p on intramuscular fat deposition in pigs based on metabolomics and transcriptomics

**DOI:** 10.1186/s12864-023-09382-6

**Published:** 2023-05-31

**Authors:** Yingke Liu, Yilin Wei, Yaqing Dou, Chenlei Li, Chenglei Song, Zhe Zhang, Kunlong Qi, Xinjian Li, Ruimin Qiao, Kejun Wang, Xiuling Li, Feng Yang, Xuelei Han

**Affiliations:** College of Animal Science and Technology, Henan Agricultural University, Zhengzhou, 450046 China

**Keywords:** miR-149-5p, Transcriptomics, Metabolomics, *ATP7A*, Porcine intramuscular preadipocytes

## Abstract

As one of the important traits in pig production, meat quality has important research significance and value. Intramuscular fat (IMF) content is one of the most important factors affecting pork quality. Many experimental studies have shown that IMF content is closely related to the flavor, tenderness, and juiciness of pork. Therefore, it is of great significance to study the mechanism of porcine IMF deposition. Previous research indicated that miR-149-5p promoted the proliferation of porcine intramuscular (IM) preadipocytes and decreased their ability to differentiate, albeit the exact mechanism of action is unknown. In vitro, foreign pigs showed increased miR-149-5p expression and reduced fat deposition when compared to Queshan Black pigs. This study conducted metabolomics and transcriptomics analyses of porcine IM preadipocytes overexpressing miR-149-5p to verify their effects on lipid formation. According to metabolomics analysis, the overexpression of miR-149-5p has significantly altered the lipid, organic acid, and organic oxygen metabolites of porcine IM preadipocytes. Specially speaking, it has changed 115 metabolites, including 105 up-regulated and 10 down-regulated ones, as well as the composition of lipid, organic acid, and organic oxygen metabolism-related metabolites. RNA-seq analysis showed that overexpression of miR-149-5p significantly altered 857 genes, of which 442 were up-regulated, and 415 were down-regulated, with enrichment to MAPK, IL-17, PI3K-Akt, and ErbB signaling pathways. We found that overexpression of miR-149-5p inhibited adipogenic differentiation by changing cAMP signaling pathway in porcine IM preadipocytes. In addition, the overexpression of miR-149-5p may affect the transport of Cu^2+^ by targeting *ATP7A* and inhibiting adipogenic differentiation. These findings elucidate the regulatory function of miR-149-5p in porcine IM preadipocytes, which may be a key target for controlling pork quality.

**Supplementary Information:**

The online version contains supplementary material available at 10.1186/s12864-023-09382-6.

## Introduction

Pork is an important source of animal protein, energy, and iron for humans as well as the economic backbone of animal husbandry [1]. An increasing number of studies show that pork currently accounts for more than 40% of all meat products consumed worldwide [2, 3]. Because of improving living standards, people now have more stringent demands for pork quality. Thus, improving the quality of pork is the top priority for the pig industry. In essence, the growth of fat and muscle is related to the breed of pig. Pork quality is closely related to the amount of intramuscular fat (IMF) [4–6]. Many experimental studies have demonstrated that IMF content is closely related to the flavor, tenderness, and juiciness of pork [7–9]. The fat breakdown has a considerable impact on the flavor of meat products. During processing, it can decompose into volatile compounds such as aliphatic hydrocarbons, aldehydes, ketones, alcohols, carboxylic acids, and esters. These chemicals can directly affect the flavor of meat products through volatilization as well as indirectly through mixing these chemicals with other compounds to produce other flavor substances [10]. IMF is mainly distributed in perimysium and endomysium. With the rise in IMF content, the number of muscle fibers per unit area will decrease, thus affecting the tenderness of meat products [11]. Pork quality is a complex trait, which is influenced by genetic factors, environment, management, feeding and slaughter conditions [12]. Therefore, understanding the mechanisms underlying animal fat deposition is essential for improving meat quality.

Preadipocytes develop into white adipocytes, which serve as both humans’ and animals’ main fuel for storing energy. Adipose tissue, one of the largest and busiest organs in both humans and animals, is essential for maintaining energy balance [13]. The two primary mechanisms that contribute to the development of adipose tissue are hypertrophy (differentiation) and adipocyte proliferation [14, 15]. The development of preadipocytes is controlled by several important genes, including lipin 1 (*LPIN1*) [16], fatty acid binding protein 4 (*FABP4*) [17], and peroxisome proliferator-activated receptor gamma (*PPARG*) [18]. The primary source of IMF is fibrous adipogenic progenitor cells (FAPs), which can differentiate into adipogenesis and fibrosis. More than 90% of FAPs in mice can differentiate into different forms of fat, and when muscles are damaged, this number decreases [19]. However, only 30% of human FAPs can form fat [20]. Therefore, learning more about the differentiation of porcine IM preadipocytes can help us better understand animal development and eventually, improve meat quality.

Approximately 22-nucleotide non-coding single stranded RNA molecules known as microRNAs (miRNAs) regulate the growth of adipose tissue, lipid metabolism, and adipogenesis in many animals [21–26]. Mounting data suggest that miRNA is crucial to the development of porcine adipocytes [27–30]. The miRNA typically binds to the 3’ untranslated region (3’UTR) sequence of its mRNA target to degrade or inhibit the process of translation [31], but it can also bind to the unconventional target of the 5’ untranslated region (5’UTR) and CDS to prevent the process of translation [32]. Numerous studies have shown that several miRNAs, such as miR-122 [33], miR-335 [34], miR-27 [35], miR-370 [36], and miR-30 [37], act as important regulators of lipid metabolism and fat deposition. A novel miRNA, which is called miR-149, has been found in the phosphatidylinositol glypican 1 (*GPC1*) gene’s first intron. Its precursor can produce the miR-149-5p and miR-149-3p active chains, which interact with target genes to perform their biological effects. Mohamed’s study found that miR-149 inhibited *PARP-2 *expression through the *SIRT-1/PGC-1* network and promoted mitochondrial biogenesis in skeletal muscle [38]. By targeting *FTO*, miR-149-3p controls the shift of bone marrow mesenchymal stem cells from adipogenesis to osteogenesis [39]. MiR-149 controls non-alcoholic fatty liver disease by targeting *FGF-21* [40]; it also inhibits the *ATF6* pathway to reduce inflammation and apoptosis in nonalcoholic fatty liver disease brought on by endoplasmic reticulum stress [41]. According to Liu et al., miR-149-5p reduces the differentiation of porcine IM preadipocytes and 3T3-L1 cells while promoting their growth [42]. However, it is still unclear how miR-149-5p influences the development of porcine IM preadipocyte. In this experiment, we conducted metabolomics and transcriptomics sequencing analysis on the overexpression of miR-149-5p.

In previous studies, we identified the role of miR-149-5p in porcine IM preadipocytes and 3T3-L1 cells. The results showed that overexpression of miR-149-5p significantly inhibited the adipogenic differentiation of porcine IM preadipocytes and 3T3-L1 cells while interference with miR-149-5p significantly promoted its adipogenic differentiation. However, the regulatory and metabolic roles of miR-149-5p in porcine IM preadipocytes remain unclear. To this end, this study aims to investigate the metabolism and transcription mechanism of miR-149-5p in porcine IM preadipocytes, the regulatory role of miR-149-5p in porcine IM preadipocytes differentiation, as well as the effect and mechanism of miR-149-5p on pork quality.

## Results

### Breed-specific differences in the expression of miR-149-5p

**Fig. 1:**
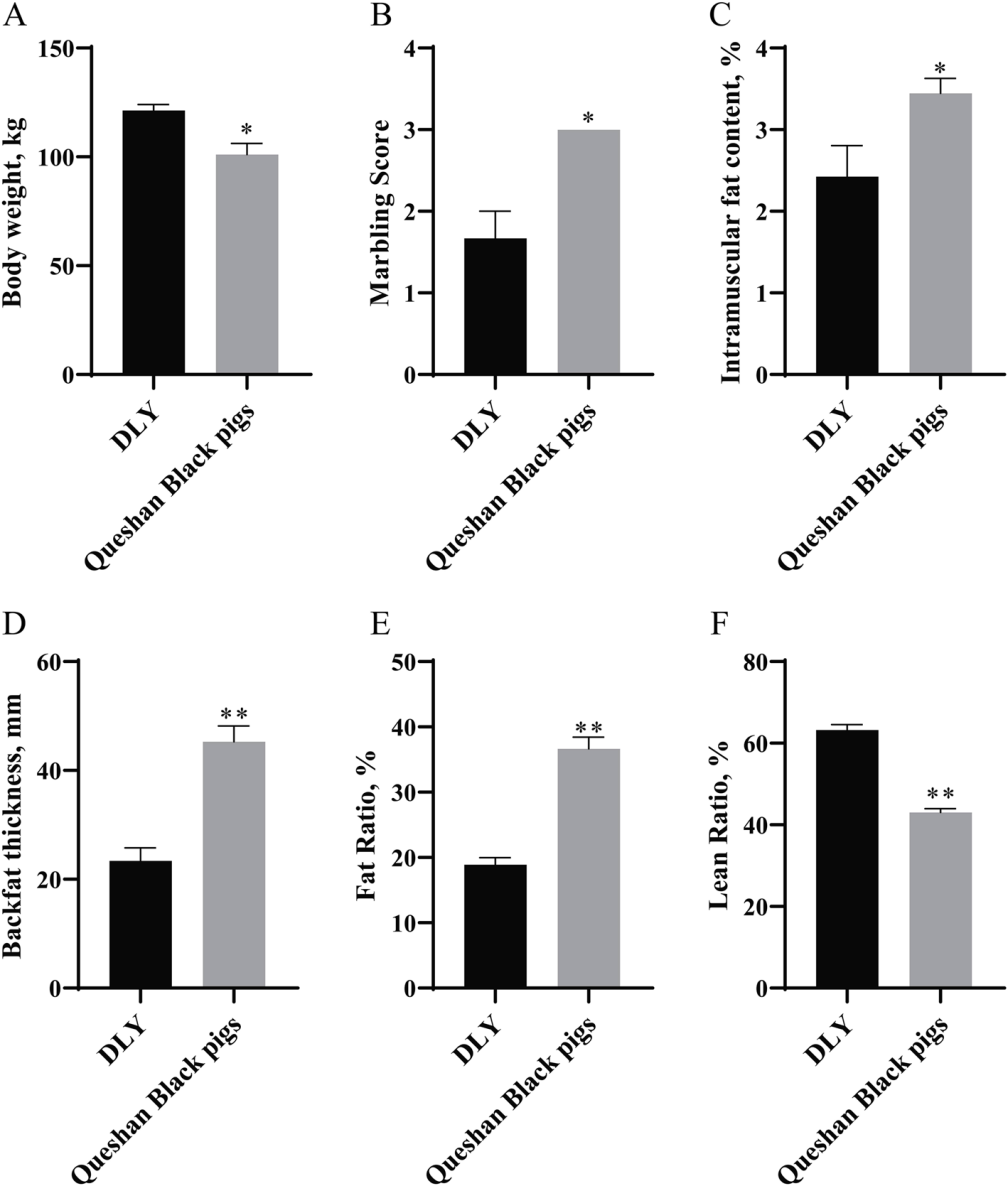
The slaughter shape of DLY and Queshan Black pigs (*n* = 3). Body weight (**A**), Marbling Score (**B**), IMF content (**C**), Backfat thickness (**D**), Fat Ratio (**E**), and Lean Ratio (**F**) of DLY and Queshan Black pigs

**Fig. 2:**
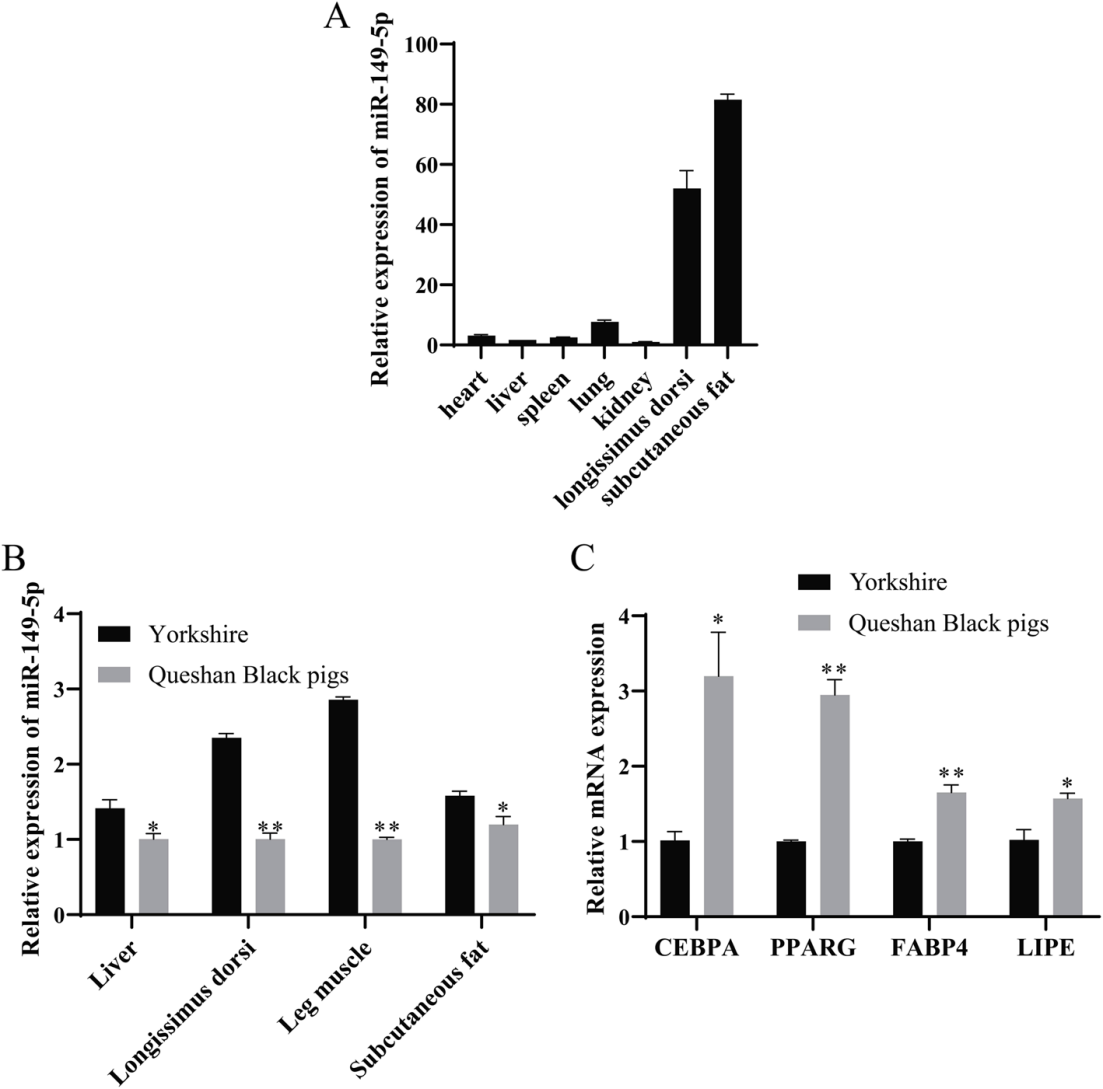
Expression patterns of Yorkshire and Queshan Black pigs (*n* = 3). **A** Tissue expression profile of miR-149-5p in Queshan Black pigs. **B** The liver, longissimus dorsi, leg muscles, and subcutaneous fat of Yorkshire and Queshan Black pigs indicate miR-149-5p expression levels. **C** The expression level of the fat deposition marker genes in the Yorkshire and Queshan Black pigs’ longissimus dorsi

The study compared the slaughter characteristics of (Duroc×Landrace×Yorkshire) DLY and Queshan Black pigs. Vitro studies showed that compared with DLY, Queshan Black pigs had lower body weight at the same age (*P* < 0.05, Fig. 1A), higher marbling score (*P* < 0.05 Fig. 1B), higher IMF content (*P* < 0.05, Fig. 1C), higher backfat thickness (*P* < 0.01, Fig. 1D), higher fat ratio (*P* < 0.01, Fig. 1E), and lower lean ratio (*P* < 0.01, Fig. 1F). The fat deposition capacity of Queshan Black pigs was significantly higher than that of DLY, and the lean ratio was significantly lower than that of DLY (*P* < 0.01). The tissue expression profile of Queshan Black pigs showed that miR-149-5p had the highest expression in subcutaneous fat, followed by longissimus dorsi (Fig. 2A). Compared with Yorkshire, the expression of miR-149-5p in the liver, longissimus dorsi, leg muscle, and subcutaneous fat of Queshan Black pigs decreased significantly (*P* < 0.05 Fig. 2B). In addition, expressions of adipogenic differentiation marker genes (*CEBPA*, *PPARG*, *FABP4*, and *LIPE*) of the longissimus dorsi in Queshan Black pigs were significantly higher than those in Yorkshire (*P* < 0.05 Fig. 2C).

### MiR-149-5p overexpression altered total metabolites in porcine IM preadipocyte

**Fig. 3:**
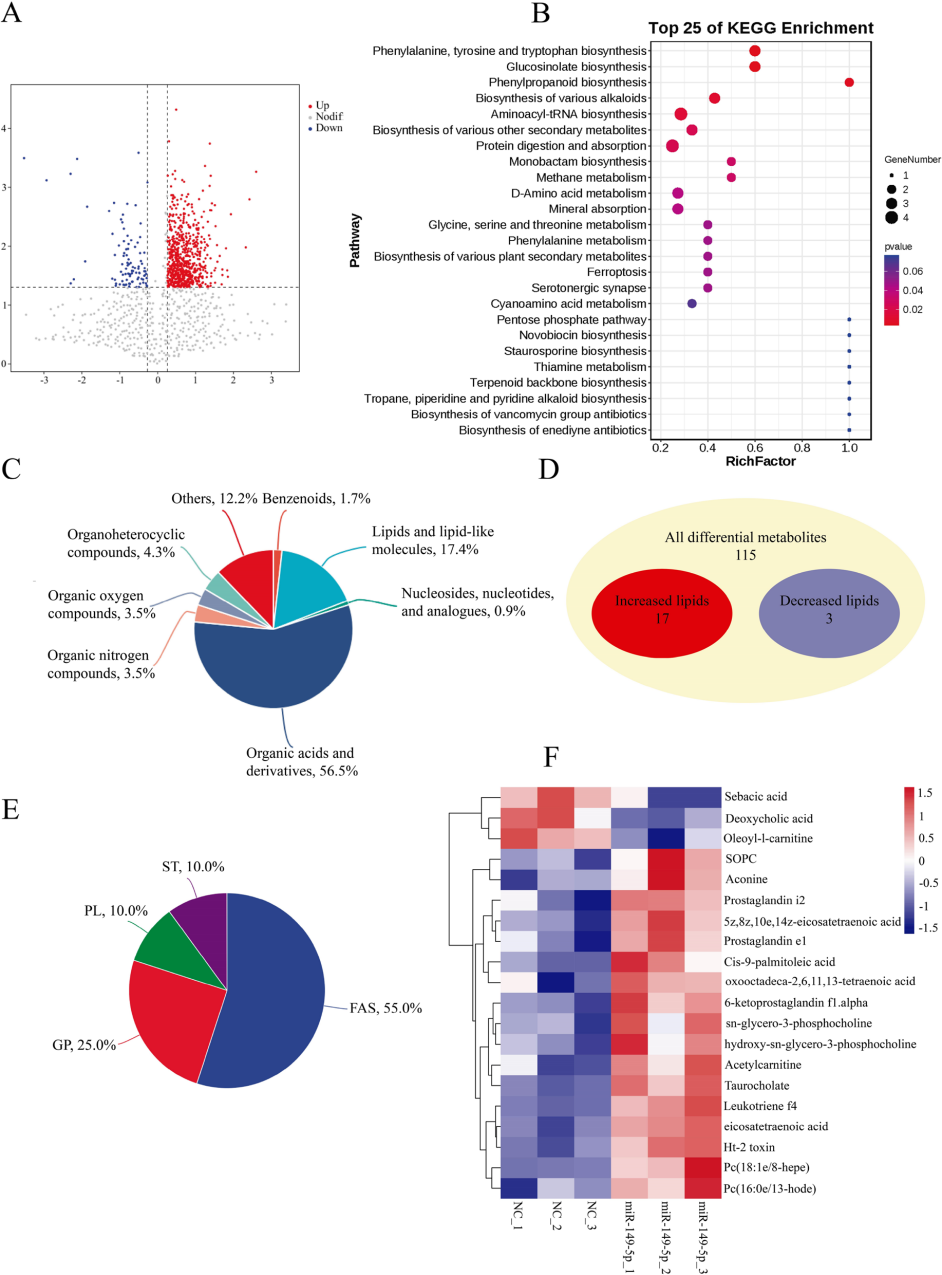
The composition of metabolites was changed by overexpression of miR-149-5p. MiR-149-5p and NC porcine IM preadipocytes were collected after 8 days of differentiation and analyzed by LC-MS/MS. **A** Volcano plot of all measured ions’ pairwise comparisons. **B** Significantly differential metabolisms were examined using KEGG pathway enrichment scatter plot statistical analysis. **C** The composition of the metabolites underwent significant change. **D** Overexpression of miR-149-5p in porcine IM preadipocytes changes lipid metabolisms. **E** The significantly changed lipid metabolites subclasses. ST, steroid; GP, glycerophospholipid; FAS, fatty acyls; PL, prenol lipid. **F** The heatmap showed the relative expression of lipid-related metabolites in the metabolic group

To investigate the effect of miR-149-5p on metabolism, we transfected porcine IM preadipocytes with miR-149-5p mimics. A total of 115 differential metabolites were found, of which 10 were down-regulated and 105 were up-regulated (Fig. 3A). KEGG results showed that the differential metabolites were highly enriched to glucosinolate biosynthesis, phenylpropanoid biosynthesis, methane metabolism, and other signal pathways (Fig. 3B). We categorized 115 metabolites according to data from the Human Metabolome Database and LIPID MAPS to comprehend the molecular type of metabolites. The amount and composition of metabolites associated with organic acids, their derivatives, and lipid metabolism showed significant alterations in cells overexpressing miR-149-5p (Fig. 3C). Among the metabolites, organic acid and derivatives metabolites (56.5%), lipid and lipid-like molecules metabolites (17.4%), nucleosides, nucleotides, and analogues metabolites (0.9%), organic nitrogen compounds metabolites (3.5%), organic oxygen compounds metabolites (3.5%), organoheterocyclic compounds metabolites (4.3%), and benzenoids metabolites (1.7%) in IM preadipocytes underwent significant change. 17 up-regulated and 3 down-regulated lipids were among the significantly changed lipid and lipid-like molecules metabolites (Fig. 3D), which were then divided into lipid subclasses (Fig. 3E). The majority of the significantly changed lipid metabolites in miR-149-5p overexpressed IM preadipocytes were fatty acids (FAS, 55.0%), glycerophospholipid (GP, 25.0%), prenol lipid (PL, 10.0%), and sphingolipid (ST, 10.0%). Significantly changed lipid metabolites were shown on the heatmap (Fig. 3F).

### Overexpression of miR-149-5p changed the expression of genes involved in metabolic pathways

**Fig. 4:**
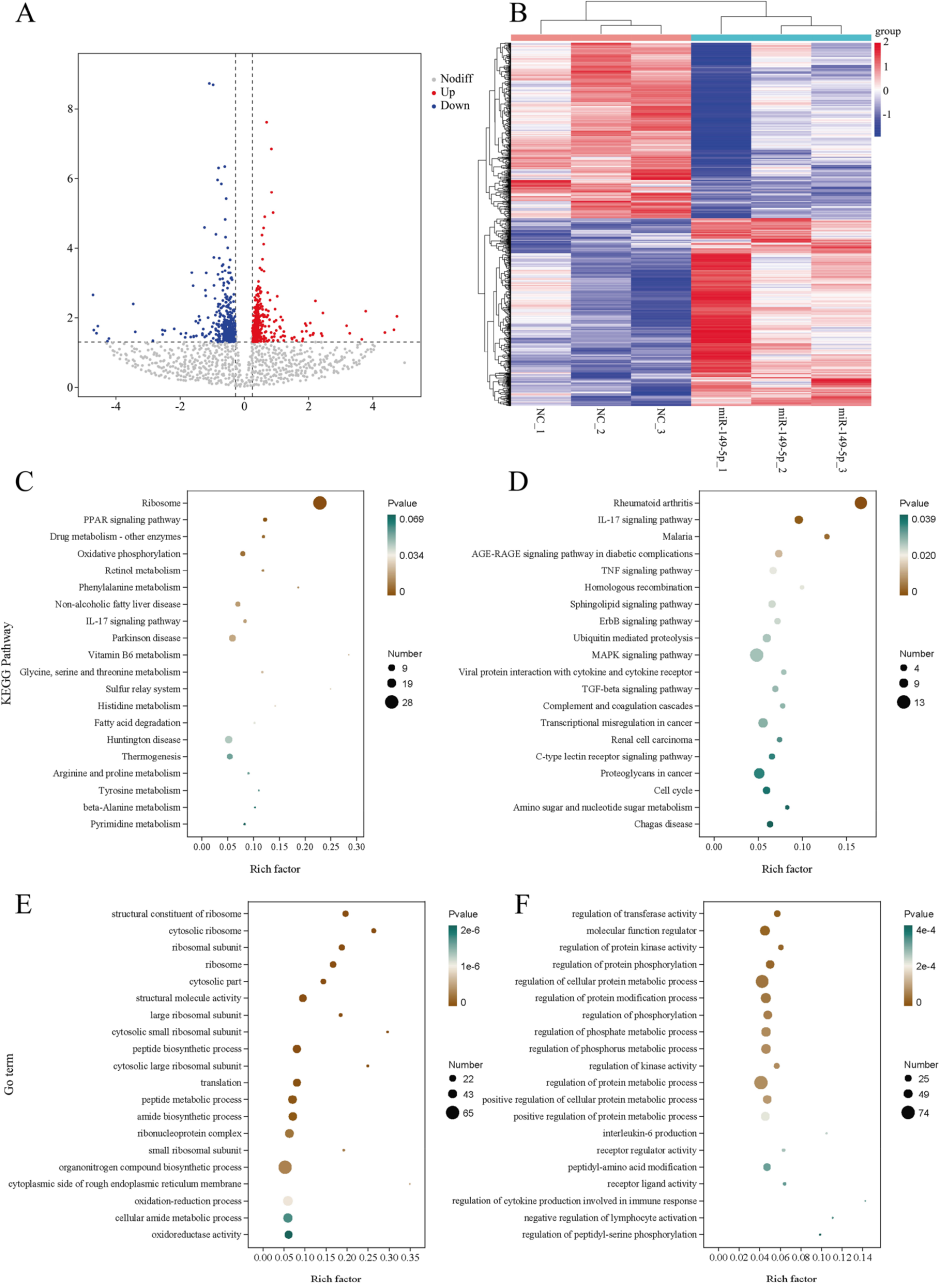
The lipid metabolism-related gene programs are activated by miR-149-5p overexpression. Porcine IM preadipocytes were collected 8 days after treatment, RNA was extracted, and RNA-seq was prepared. **A** Volcano plot of all measured genes’ pairwise comparisons. **B** The heatmap showed the relative expression of differential genes in RNA-seq. **C** Significantly up-regulated genes were examined by using KEGG pathway enrichment scatter plot statistical analysis. **D** Significantly down-regulated genes were examined by using KEGG pathway enrichment scatter plot statistical analysis. **E** Significantly up-regulated genes were examined by using GO pathway enrichment scatter plot statistical analysis. **F** Significantly down-regulated genes were examined by using GO pathway enrichment scatter plot statistical analysis

We used RNA-seq to map the transcriptional changes in adipocytes as a result of miR-149-5p overexpression to investigate how the adipocyte metabolites were changed. We found 857 differentially expressed genes, among which 442 were up-regulated, and 415 were down-regulated upon miR-149-5p overexpression (Fig. 4A). Significantly changed genes were displayed in the heatmap (Fig. 4B). Up-regulated genes in the overexpression of miR-149-5p gathered in PPAR and IL-17 signaling pathways, according to KEGG pathway analysis (Fig. 4C). In addition, down-regulated in the overexpression of miR-149-5p gathered in IL-17, MAPK, and ErbB signaling pathways (Fig. 4D). The analysis of differentially up-regulated genes revealed a significant increase in the process of peptide biosynthetic and amide biosynthetic (Fig. 4E). The analysis of differentially down-regulated genes revealed a significant decrease in regulation of protein kinase activity, protein phosphorylation, and interleukin-6 production (Fig. 4F).

### Overexpression of miR-149-5p affected lipid metabolism of porcine IM preadipocytes

**Fig. 5:**
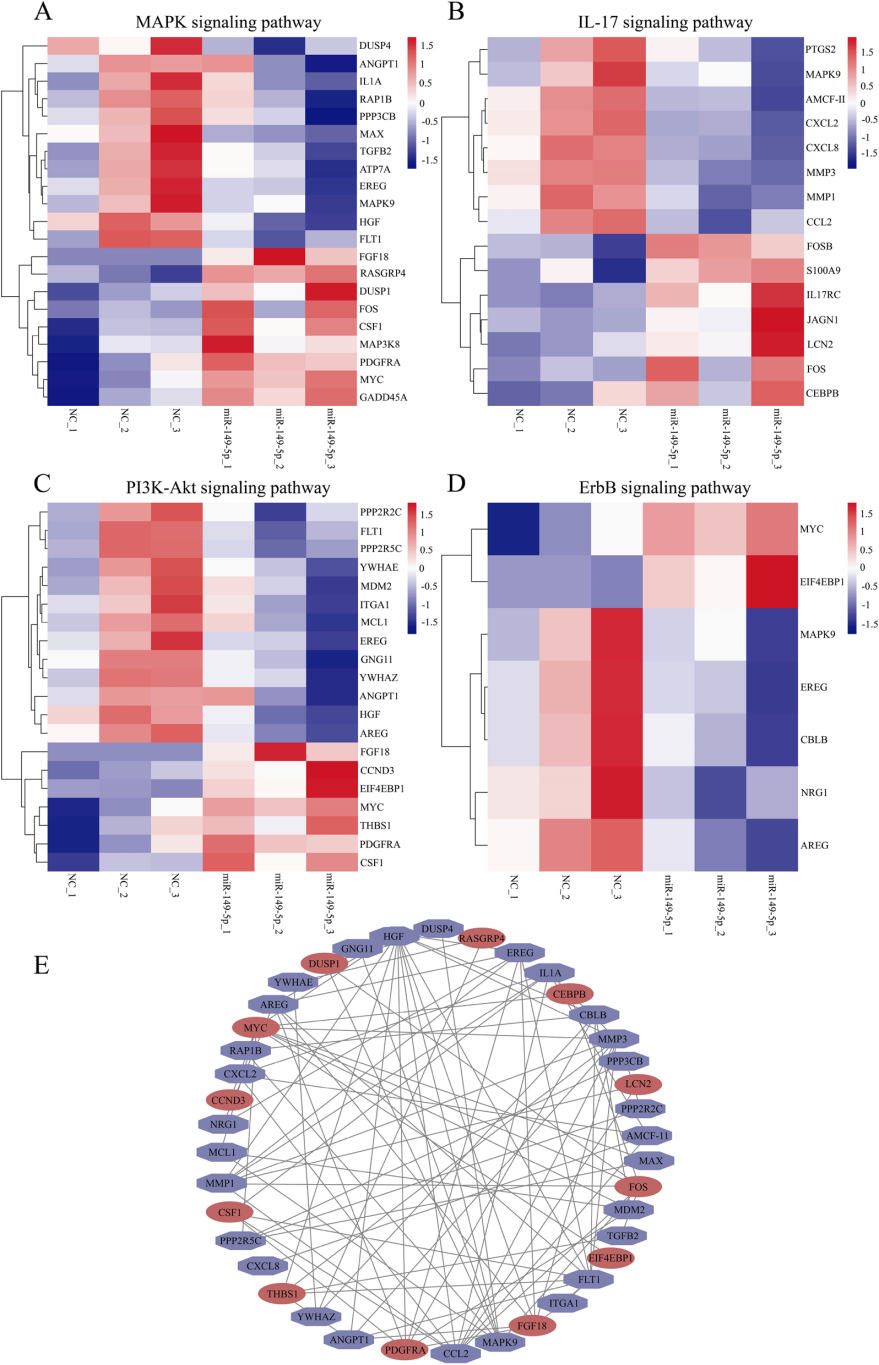
Overexpression of miR-149-5p regulated lipid-related metabolic pathways. Heatmaps showing the picked DEGs related to MAPK (**A**), IL-17 (**B**), PI3K-Akt (**C**), and ErbB signaling pathway (**D**) in porcine IM preadipocytes overexpressing miR-149-5p. **E** Protein-Protein Interaction Network (PPI) of MAPK, IL-17, PI3K-Akt, and ErbB signaling pathway-related genes

**Fig. 6:**
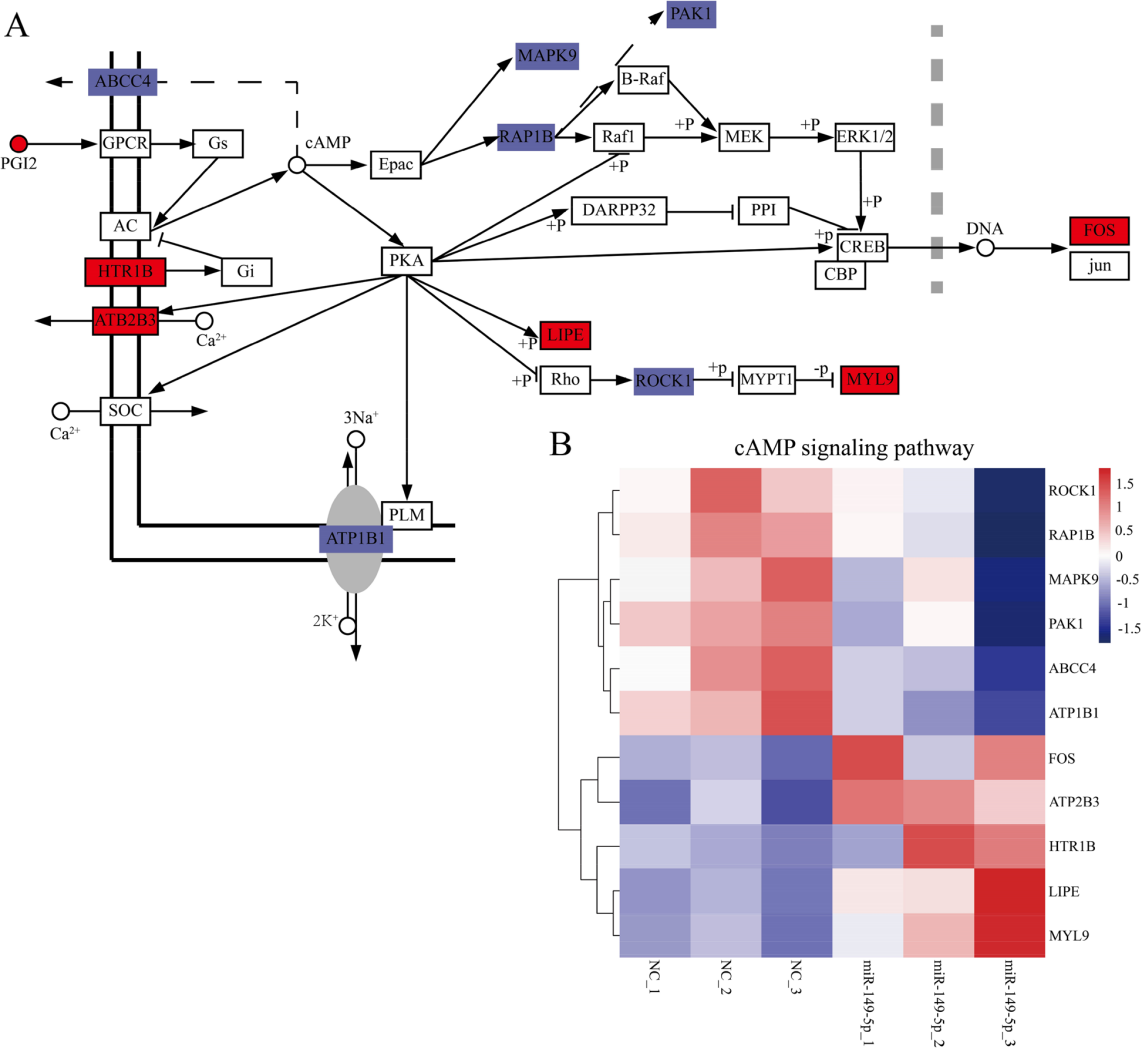
Overexpression of miR-149-5p regulated cAMP signaling pathway. **A** KEGG analysis of cAMP signaling pathway-related genes showed that the metabolic response of porcine IM preadipocytes was significantly regulated after miR-149-5p overexpression. Red indicated up-regulation and blue indicated down-regulation. **B** Heatmap showed the picked DEGs related to cAMP signaling pathway in porcine IM preadipocytes overexpressing miR-149-5p

To determine the signal pathway involved in lipid metabolism, we used bioinformatics analysis methods to select metabolite-related genes from the KEGG pathway (Fig. 5A–D). Heatmap demonstrates that the miR-149-5p overexpression dramatically altered the expression of the followings: MAPK signal pathway-related genes, including interleukin 1 alpha (*IL1A*), ATPase copper transporting alpha (*ATP7A*), and epiregulin (*EREG*) (Fig. 5A); IL-17 signal pathway-related genes, including prostaglandin-endoperoxide synthase 2 (*PTGS2*), mitogen-activated protein kinase 9 (*MAPK9*), interleukin 17 receptor C (*IL17RC*), and CCAAT enhancer binding protein beta (*CEBPB*) (Fig. 5B); PI3K-Akt signal pathway-related genes, including fms related receptor tyrosine kinase 1 (*FLT1*), hepatocyte growth factor (*HGF*), and colony stimulating factor 1 (*CSF1*) (Fig. 5C); and ErbB signal pathway-related genes, including MYC proto-oncogene, bHLH transcription factor (*MYC*), cbl proto-oncogene B (*CBLB*), and amphiregulin (*AREG*) (Fig. 5D). We performed protein-protein interaction analysis on the genes in these four pathways and found that 40 genes had significant protein-protein interaction, especially (chemokine (C-C motif) ligand 2) *CCL2*, (mitogen-activated protein kinase 9) *MAPK9*, (matrix metallopeptidase 3) *MMP3*, and *HGF* (Fig. 5E). We constructed a cAMP signaling pathway metabolic network to describe how differentially expressed genes (DEGs) mediate the regulation of miR-149-5p (Fig. 6A). In addition, we performed a heatmap clustering analysis of cAMP signaling pathway-related genes (Fig. 6B).

### MiR-149-5p influenced lipid formation by targeting *ATP7A*

**Fig. 7:**
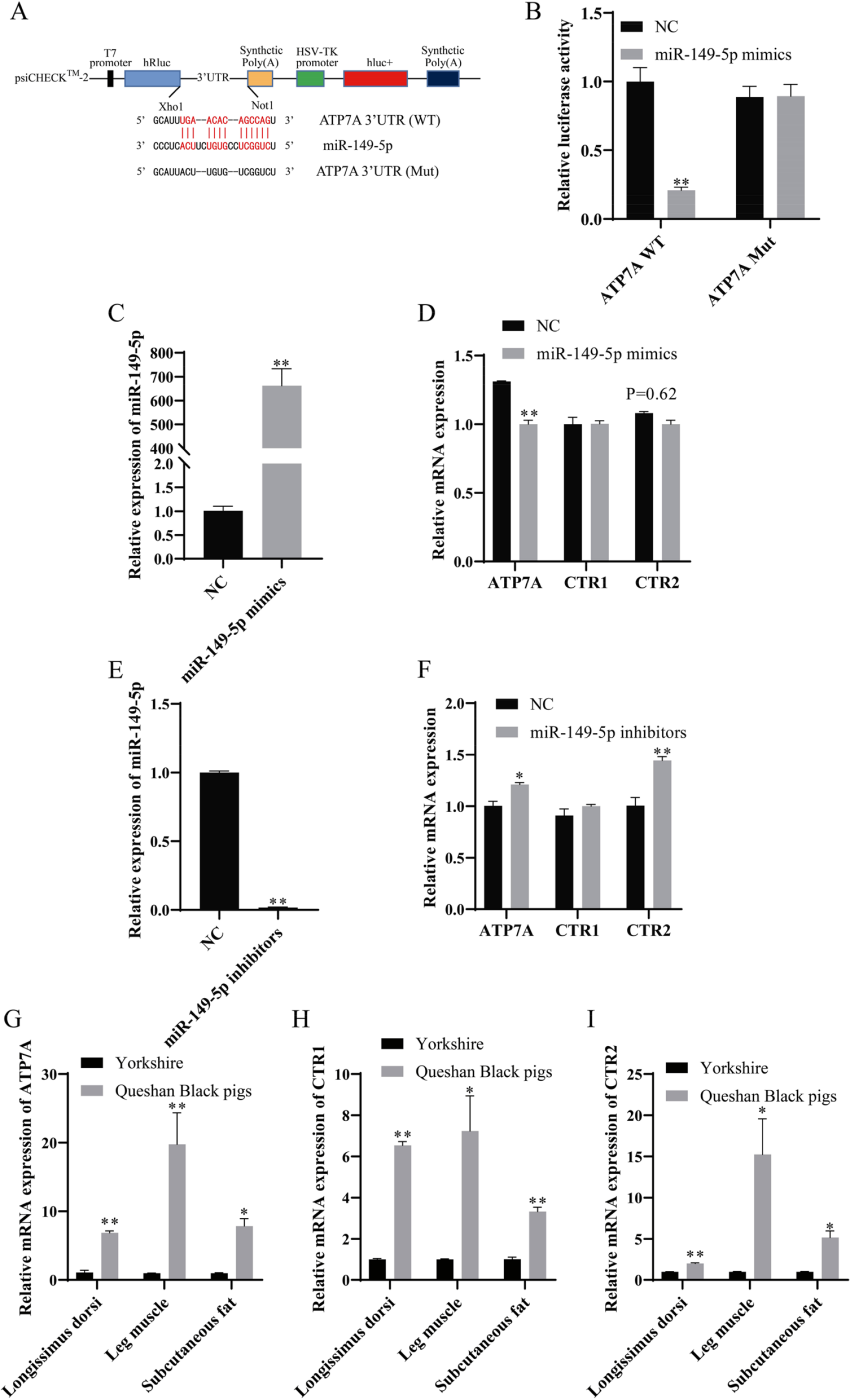
MiR-149-5p targets *ATP7A* and affects IMF formation. **A** Schematic construction of *ATP7A* 3′ UTR dual-luciferase reporter vector. **B** Dual-luciferase assay detected porcine *ATP7A* in 293T cells. **C** After injecting miR-149-5p mimics into the porcine IM preadipocytes, miR-149-5p expression was observed (*n* = 3). **D** After 8 day’s induction, *ATP7A*, *CTR1*, and *CTR2* in the porcine IM preadipocytes were checked (*n* = 3). **E** After injecting miR-149-5p inhibitors into the porcine IM preadipocytes, miR-149-5p expression was observed (*n* = 3). **F** After 8 day’s induction, *ATP7A*, *CTR1*, and *CTR2* in the porcine IM preadipocytes were checked (*n* = 3). **G**–**I** The relative expressions of *ATP7A* (**G**), *CTR1* (**H**), and *CTR2* (**I**) in the longissimus dorsi, leg muscles, and subcutaneous fat of Queshan Black pigs in comparison with Yorkshire (*n* = 3)

According to the results of RNA-seq, once miR-149-5p was overexpressed, the expression of *ATP7A* drastically dropped, and since RNA22 v2 suggested that they had binding sites (Fig. 7A), it was assumed that *ATP7A* was miR-149-5p’s target. To prove that, dual-luciferase assay detection was done on wild-type and mutant vectors. The findings showed that miR-149-5p significantly decrease the activity of the wild-type vector without having impact on the activity of the mutant vector when compared to the control group (*P* < 0.01, Fig. 7B). *ATP7A* is a Cu^2+^ transporter, which may affect the formation of IMF by affecting the transport of Cu^2+^. MiR-149-5p expression was considerably enhanced upon transfection (*P* < 0.01, Fig. 7C). MiR-149-5p mimics dramatically reduced the expression of *ATP7A* in porcine IM preadipocytes (*P* < 0.01), and the expressions of Cu^2+^ transporters *CTR1* and *CTR2* but insignificantly (Fig. 7D). To detect the targeting of miR-149-5p and *ATP7A*, this study interfered with miR-149-5p (Fig. 7E). MiR-149-5p inhibitors significantly increased the expression of *ATP7A* and *CTR2* in porcine IM preadipocytes (*P* < 0.05), and the expression of Cu^2+^ transporter *CTR1* but insignificantly (Fig. 7F). Meanwhile, compared with foreign pigs, Queshan Black pigs showed significantly increased expressions of *ATP7A*, *CTR1*, and *CTR2* in the longissimus dorsi, leg muscles, and subcutaneous fat (Figs. 7G–I).

## Discussion

MiR-149-5p regulates energy metabolism in addition to genes associated with fat storage and obesity. We summarized the metabolic and transcriptional characteristics of porcine IM preadipocyte overexpressed with miR-149-5p. Furthermore, this experiment found that miR-149-5p may affect the transport of Cu^2+^ by targeting *ATP7A*, further affecting the formation of IMF. Phospholipids are significant compounds that impact the taste of meat products, making up IMF together with triglycerides. They are mainly composed of C:16 and C:18 fatty acids [43]. Pork quality is determined by the kind and percentage of fatty acids, and this can serve as a benchmark when judging the quality of other meats [44]. Monounsaturated and saturated fatty acids can improve the quality of meat, whereas polyunsaturated fatty acids have the opposite effect [45]. IMF is also closely related to the tenderness of meat products. It not only destroys the cross-linked structure between muscle fiber bundles but also weakens the strength of connective tissue, thus reducing the shearing force of muscle and improving the tenderness of meat products [46].

Having a long history and a sizable population in Queshan County, Henan Province, the Queshan Black pig is a superior local breed of pigs. They are characterized by strong fecundity, strong adaptability, good meat quality, and stable genetic performance [47–49]. In vitro, studies showed that the fat deposition ability of Queshan Black pigs was significantly higher than that of foreign pigs, and the expression of miR-149-5p in subcutaneous fat was significantly decreased, indicating that the expression of miR-149-5p was possibly related to fat accumulation. In addition, compared with foreign pigs, miR-149-5p in Queshan Black pigs was significantly decreased in liver, longissimus dorsi, and leg muscles, indicating that miR-149-5p expression was associated with possible IMF deposition. The liver is an important metabolic organ in animals, which is closely related to lipid deposition. In previous studies, miR-149-5p overexpression inhibited the differentiation of porcine IM preadipocytes and the expression of fat formation-related genes. At the same time, many studies found that miR-149 is strongly correlated to fat production. For instance, miR-149 induces subcutaneous fat change into visceral fat and inhibits *PRDM16* [50]. In addition, the decrease of miR-149-3p significantly increased the expression of *PRDM16* in SAT, improved the systemic insulin sensitivity of high fat-fed mice, and alleviated SAT inflammation and liver steatosis [51]. In studies on cattle, the expression of miR-149-5p was substantially higher in bovine preadipocytes than in adipocytes [52]. By targeting *CRTC* genes, miR-149-5p can prevent the proliferation and differentiation of bovine adipocytes [53]. Mouse mesenchymal stem cells promote differentiation by miR-149, which targets *Dab2* [54]. Moreover, miR-149-5p inhibits melanoma cell proliferation by targeting *LRIG2* [55].

*MAPK* is a kind of serine/threonine protein kinase that widely exists in mammals and can be triggered by external signals, such as bacterial complexes, cytokines, growth factors, and physical stress. The regulation of the MAPK signaling pathway, which is connected to the regulation of a variety of cellular activities, has become one of the most active research areas in cell signal transduction in recent years. The MAPK signaling pathway can phosphorylate a wide range of substrates, including a wide range of nuclear transcription factors and protein kinases, thereby controlling the transcription of related genes; it takes part in several physiological processes, such as cell growth, development, and functional synchronization among cells; it is crucial for adipocyte differentiation, which in turn affects obesity [56]. PPI is composed of proteins that interact with each other, to participate in cell cycle regulation, energy and material metabolism, gene expression regulation, biological signal transmission, and other vital processes. *CCL2*, *MAPK9*, *MMP3*, and *HGF* have significant protein-protein interactions, which are related to adipogenesis. In vitro knockout of *CCL2* reduces macrophage migration to EMSC, thereby reducing fat content [57]. In luteolin-treated epididymal white adipose tissue, *MAPK9* gene expression and adipogenesis were found to be positively correlated [58]. *MMP3* overexpression prevents the differentiation of 3T3-L1 cells [59]. A high fat-fed fatty liver can be greatly improved by overexpressing *HGF* [60]. Transcriptomics research is an important means of functional genomics research, which studies gene expression from the level of all mRNA in a cell, and metabolomics studies the expression of cell metabolites. The combination of different omics technologies is an important strategy in the era of functional genomics, which has the characteristics of integrity and high throughput. Therefore, transcriptomics and metabolomics technologies are applied to complex metabolic studies such as obesity, and the results at different levels are integrated to obtain the exact mechanism of the occurrence of research objects. Studies on transcriptomics and metabolomics revealed that *CRTC3* overexpression promoted adipogenic differentiation by up-regulating Ca^2+^, and cAMP signaling pathway in IM and SC adipocytes [61].

*ATP7A*, also known as Menkes disease protein, is a P-type ATPase that transports Cu^2+^ across cell membranes. It is well known that lipid metabolism and copper homeostasis are closely related [62]. This experiment discovered that the miR-149-5p target gene is *ATP7A*. In the meantime, the *ATP7A* in the longissimus dorsi, leg muscles, and subcutaneous fat of Queshan Black pigs was significantly higher than that of Yorkshire, and the Cu^2+^ transporters *CTR1* and *CTR2* were also increased, which further indicated that miR-149-5p may affect the transport of Cu^2+^ by targeting *ATP7A*, thereby inhibiting the formation of IMF. The study found that cold-stimulated brown adipose tissue showed an increase in the level of Cu^2+^ and a significant increase in *ATP7A* level [63]. Tao et al. found that the level of Cu^2+^ concentration of *ATP7A* knockout mice significantly increased, and the fat content significantly decreased [64]. The differential expression of *ATP7A* in porcine IM preadipocytes possibly influences the effect of the level of Cu^2+^ on lipid metabolism in adipocytes.

## Conclusions

Based on transcriptomics and metabolomics, we studied the differences in miR-149-5p expression in adipose tissue of local and foreign pigs and revealed the effect of miR-149-5p overexpression on adipocyte metabolism. MiR-149-5p overexpression may inhibit fat accumulation in adipocytes by regulating MAPK, IL-17, PI3K-Akt, and ErbB-related signaling pathways. We discovered that Cu^2+^ transporter *ATP7A* is the target gene of miR-149-5p. MiR-149-5p may inhibit the transport of Cu^2+^ and further inhibit IMF formation by targeting *ATP7A*. This study provides the first detailed analysis and fresh perspectives of how miR-149-5p overexpression affects the metabolism and transcription of porcine IM preadipocytes and enhances our comprehension of the molecular characteristics of fat and energy metabolism regulated by miR-149-5p. And the results of this study provide a theoretical formation for improving pork quality traits.

## Materials and methods

### Experimental animals and sample collection

Three Queshan Black pigs and three DLY at a weight of about 110 kg were randomly selected for vitro experiments to study the expression pattern of miR-149-5p and its relationship with fat deposition in pigs. These pigs were fed at the same nutritional level and under the same management conditions. Before being slaughtered, the pigs were required to fast for 24 hours while being given free access to water. After slaughter, the lean and fat weights of the pigs were measured, and the lean and fat meat rates were calculated. We used a Vernier caliper to measure the thickness of the three-point backfat. Marbling was scored by using the US NPPC flesh color comparison board. And the IMF content of the longissimus dorsi was measured by using an extraction method.

### Cell culture and transfection

Porcine IM preadipocytes were isolated from 3-day-old Queshan Black piglets by using the published method [42]. The improved Eagle medium contains 89% Durbeko (Gibco, Carlsbad, USA), 10% FBS (Gibco, Carlsbad, USA), and 1% penicillin-streptomycin (Solarbio, Beijing, China) growth medium to cultivate porcine IM preadipocytes. Cells were inoculated into 6-well plates and transfected with Lipofectamine 3000 (Thermo Fisher Scientific, USA) when the cell density reached 70–80%.

### RNA extraction and reverse transcription

RNA extraction operation steps refer to the instructions of Trizol reagent (Thermo Fisher Scientific, USA), and RNA concentration was measured by the nucleic acid detector. RNA was reverse transcribed into cDNA by a reverse transcription kit (Accurate Biology, Hunan, China).

### qRT-PCR

Adipogenic marker genes such as *CEBPA*, *PPARG*, *FABP4*, and *LIPE* were selected to identify the adipogenic differentiation ability of the longissimus dorsi in Queshan Black and Yorkshire. *ATP7A* was selected to verify the reliability of RNA-seq. The target genes were quantitatively detected by CFX96 Real-time PCR instrument. The primers were synthesized by Shanghai Sangon Biotech Company. Based on the internal reference gene *GAPDH*, the expression of the target genes in each template was calculated by 2^-ΔΔCt^ method. At the same time, the expression of miR-149-5p takes U6 (RiboBio, Guangzhou, China) as an internal reference.

### Dual-luciferase reporter assay

To figure out if miR-149-5p targets *ATP7A*, two versions of the luciferase reporter plasmid vector (psi-CHECK2) were made: one with a wild-type 3′UTR (WT-*ATP7A*) and the other with a mutant 3′UTR (Mut-*ATP7A*). Following that, miR-149-5p mimics and psiCHECK2-*ATP7A*-3′UTR were cotransfected into 293T cells, the control group was used instead. After lysing the cells, the firefly luciferase activities were found after 48 hours’ transfection by using a luciferase reporter assay kit (Promega, Madison, WI, U.S.A.).

### RNA-seq analysis

After 8 days of differentiation, miR-149-5p and NC cells were collected, treated with Trizol reagent, stored on dry ice, and sent to Shanghai Personal Bio Company (Shanghai, China) for RNA-seq. The first cDNA was synthesized by using random oligonucleotides and Super Script II. Subsequently, the second strand cDNA was synthesized by using DNA polymerase I and RNase h, and the remaining dangling was converted to a blunt end by exonuclease/polymerase activity and the enzyme was removed. After adenylation at the 3’ end of the DNA fragment, the Illumina PE aptamer oligonucleotide was ligated to prepare for hybridization. In order to select the optimal cDNA fragment with a length of 400–500 bp, the library fragment was purified by using the AMPure XP system (Beckman Coulter, Beverly, CA, USA). Agilent 2100 Bioanalyzer (Agilent, 2100) and Agilent High Sensitivity DNA Kit (Agilent, 5067 − 4626) were used to detect the quality of the library. After homogenization, multiplexed DNA libraries were mixed in equal volumes. The mixed library was gradually diluted and quantified, and PE150 mode sequencing was performed on the Illumina sequencer. After the sample was sequenced on the computer, the image file was obtained, and the original data (Raw Data) of FASTQ was generated by the software of the sequencing platform. Download the reference genome and gene model annotation files directly from the genome website. An index of the reference genome is constructed by using HISAT2v2.0.5 and the paired-end clean reads are compared to the reference genome by using HISAT2v2.0.5. HTSeq (0.9.1) was used to compare the Read Count values on each gene as the original expression of the gene. Differential expression analysis of the two comparison combinations was performed by using DESeq2 software (Version 1.12.4). A significant change was referred to as the threshold (|log_2_ fold change| > log_2_ 1.2; *P* < 0.05). GO enrichment and KEGG analysis of DEGs were used by the Gene Denovo Cloud Platform (https://www.omicshare.com/) [65–67].

### Metabolomics analysis

After 8 days of differentiation, miR-149-5p and NC cells were collected, stored on dry ice, and sent to Shanghai Personal Bio Company (Shanghai, China) for LC-MS/MS analysis. Take each sample, make it slowly thawed at 4 °C, and add 1mL methanol: acetonitrile: water (2:2:1, v/v) for full vortex mixing, ultrasonic crushing at low temperature, and incubation for 1 h at -20 °C to precipitate protein, centrifuged at 13,000 rpm and 4 °C for 15 min, with the supernatant freeze-dried and stored at -80 °C for later use. During mass spectrometry analysis, 100 µL acetonitrile aqueous solution (acetonitrile: water = 1:1, v/v) was added for reconstitution, vortexed, and centrifuged at 14000 g and 4 °C for 15 min. Use its company website for drawing. The samples were separated by Agilent 1290 Infinity LC ultra-high performance liquid chromatography (UHPLC) HILIC column. To avoid the influence of instrument detection signal fluctuation, the continuous analysis of samples is carried out in random order. QC samples are inserted into the sample queue to monitor and evaluate the stability of the system and the reliability of the experimental data. The samples were separated by UHPLC and analyzed by Triple TOF 6600 mass spectrometer (AB SCIEX).

MiR-149-5p was compared with NC to reveal the changes of metabolites in porcine IM preadipocytes after overexpression of miR-149-5p. Orthogonal partial least squares discriminant analysis (OPLS-DA) was used for statistical analysis to figure out the changes in metabolites between groups. A significant change was referred as the threshold (|log_2_ fold change| > log_2_ 1; *P* < 0.05; VIP > 1). GO enrichment and KEGG analysis of differential metabolites was used by the Gene Denovo Cloud Platform (https://www.omicshare.com/).

### Bioinformatics analysis

The binding site of mRNA and miRNA was predicted by using the online tool RNA22 v2 (https://cm.jefferson.edu/rna22/Interactive/).

### Statistical analyses

The Student’s t-test of SPSS 26.0 software was used, and *P < 0.05 and **P < 0.01 was used as the criterion for a significant difference. The statistical data were expressed as ‘mean ± SD (*n* = 3)**’**.

## Supplementary Information

**Additional file 1.**

**Additional file 2.**

**Additional file 3.**

**Additional file 4.**

## Data Availability

All raw data of transcriptomics sequencing have been deposited to the National Genomics Data Center (NGDC, https://bigd.big.ac.cn) with the dataset accession number CRA009692 (https://ngdc.cncb.ac.cn/gsa/s/eg5Yhm4u). The metabolite data set supporting the results of this article is included within the article, and can be found in the Supplemental Information (Additional file 2: Table S2).
