# Supplementary material for: Effect of miR-149-5p on intramuscular fat deposition in pigs based on metabolomics and transcriptomics
Source: BMC Genomics. 2023 May 31;24:293. doi: 10.1186/s12864-023-09382-6 (PMC10230699; doi:10.1186/s12864-023-09382-6)
Supplement: Supplementary file 1 — Additional file 1. [file 12864_2023_9382_MOESM1_ESM.docx]

Additional file 1: Table S1. Primer information for qPCR verification

| Primer | Sequence |
| --- | --- |
| GAPDH-F | GCCAAAAGGGTCATCATCTC |
| GAPDH-R | GTAGAGGCAGGGATGATGTTC |
| miR-149-5p-F | CGTCTGGCTCCGTGTCTTC |
| miR-149-5p-R | AGTGCAGGGTCCGAGGTATT |
| CEBPA-F | ACACGGTGCGTCTAAGATGAGG |
| CEBPA-R | TCGGAGCGGTGAGTTTGC |
| PPARG-F | AAATGCCTTGCTGTGG |
| PPARG-R | GCCCTCGCCTTTGCTT |
| FABP4-F | CCAAACCCAACCTGAT |
| FABP4-R | CATGATACATTCCACCAC |
| LIPE-F | ACGAAGGCGTCACTGCTG |
| LIPE-R | TTTGTAATGCTCCCCGAA |
| ATP7A-F | ATTCTGTACTTGTCGCTC |
| ATP7A-R | ATTCCTCTGTGTTTTGTG |
| CTR1-F | TCATCATCACCCAACCTC |
| CTR1-R | AATCACCAAACCAGCAAA |
| CTR2-F | CAGACAGCAGGACCCATC |
| CTR2-R | ACCACGCCAAAGAAAATC |
