# Supplementary material for: Effect of miR-149-5p on intramuscular fat deposition in pigs based on metabolomics and transcriptomics
Source: BMC Genomics. 2023 May 31;24:293. doi: 10.1186/s12864-023-09382-6 (PMC10230699; doi:10.1186/s12864-023-09382-6)
Supplement: Supplementary file 4 — Additional file 4. [file 12864_2023_9382_MOESM4_ESM.docx]

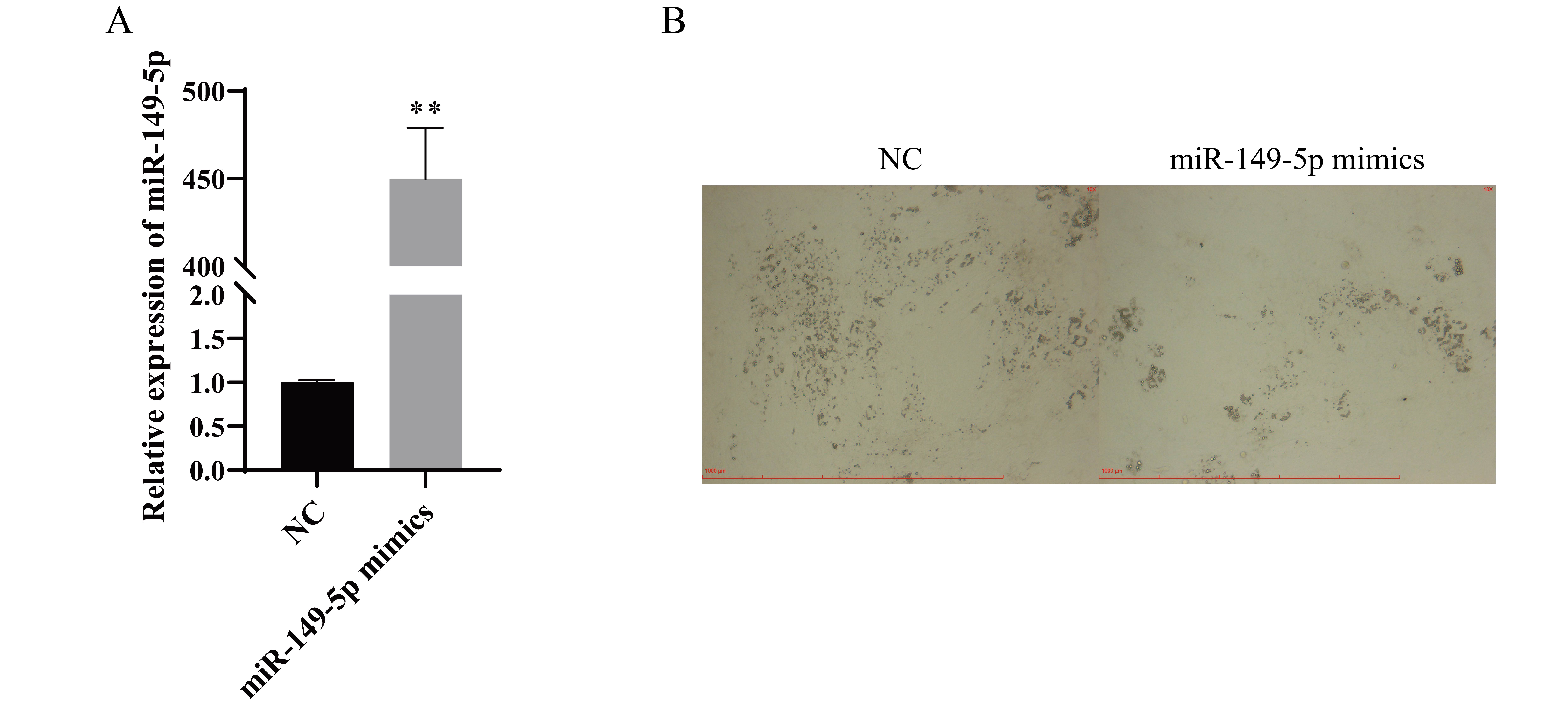


**Table S4.** Quality control before omics analysis. (A) After injecting miR-149-5p mimics into the porcine IM preadipocytes, miR-149-5p expression was observed (n=3). (B) After injection of miR-149-5p mimics into porcine IM preadipocytes, the cell morphology was observed after 8 d of differentiation.
